# Spatially heterogeneous and nonlinear factors influencing intercity patient mobility for chronic kidney disease: a nationwide study in China

**DOI:** 10.1038/s44401-026-00086-z

**Published:** 2026-05-18

**Authors:** Chenghua Guo, Jingyi Wu, Qianlin Zuo, Pengfei Li, Luxia Zhang

**Affiliations:** 1 School of Nursing, Peking University, Beijing, China; 2 Advanced Institute of Information Technology, Peking University, Hangzhou, China; 3 PKU-WUHAN Institute for Artificial Intelligence, Wuhan, China; 4 National Institute of Health Data Science, Peking University, Beijing, China; 5 Renal Division, Department of Medicine, Peking University First Hospital; Peking University Institute of Nephrology, Beijing, China; 6 Institute for Artificial Intelligence, Peking University, Beijing, China

**Keywords:** Health care, Nephrology

## Abstract

Uneven distribution of high-quality nephrology care in China has driven rising intercity patient mobility for chronic kidney disease (CKD). This study examined the spatial correlates of this mobility using over 4 million cross-city hospitalization records from 2014 to 2018. First, the Geodetector model was used to identify the key factors and their complex interactions driving patient inflows and outflows, including socioeconomic status, healthcare resource availability, and transportation accessibility. Then, multiscale geographically weighted regression (MGWR) was applied to explore geographical heterogeneity in the influence of these factors on intercity patient mobility. According to the Geodetector model, the leading correlates of patient outflows included hospital bed density, doctor density, and population growth rate, with evident nonlinear and synergistic associations. Patient inflows were mainly influenced by nephrology workforce availability and population structure. MGWR analysis revealed substantial spatial variation in the associations of general and nephrology-specific healthcare resources on intercity patient mobility, underscoring the complex interaction between healthcare capacity and geographic context. This study proposes a novel framework for understanding the spatial correlates of intercity CKD patient mobility in China and highlights the geographic heterogeneity of their associations. The findings support policies aimed at improving the equity and efficiency of CKD care across regions.

## Introduction

Chronic kidney disease (CKD) is a progressive, long-term condition that poses a growing public health challenge in China. In recent decades, the burden of CKD in China has risen sharply, driven by increasing rates of diabetes, hypertension, obesity, and an aging population^1^. As of 2019, the estimated prevalence of CKD in China reached 8.2%, with public awareness remaining alarmingly low at around 10%^2^. CKD is associated with serious complications, including cardiovascular disease, renal failure, and increased mortality^3^, placing a heavy burden on both patients and the healthcare system. Effective management of CKD requires ongoing, specialized care^4^. Despite growing demand for quality kidney care, there remains a substantial gap between the need for kidney care services and the resources and capacity of the healthcare system, particularly in less developed regions^5–7^. Previous evidence showed that the prevalence of CKD is higher in the northern and southwestern regions^8^, whereas one-third of the country’s nephrologists are concentrated in East China^7^.

Against this backdrop, intercity mobility of CKD patients has emerged as a notable phenomenon, driven largely by the uneven distribution of high-quality medical services. The lack of rigid regulation on patient referral and the introduction of immediate cross-city reimbursement policies have made it easier for patients to seek care beyond their home regions^9^. Cross-city visits in China surged from 65.32 million in 2018 to 110.5 million in 2022^10,11^. Patients are motivated to access cross-city healthcare due to inadequate local services or in pursuit of better care options. In 2022, non-local patients accounted for nearly 40% of tertiary inpatients in major hubs like Beijing and Shanghai^12^. Although this mobility can improve access and outcomes, it also increases healthcare costs, heightens the travel burden for patients^13^, and places added pressure on top-tier hospitals, potentially exacerbating inequalities and undermining efforts to build a sustainable, tiered healthcare delivery system^14,15^.

In response, national healthcare reforms have begun to focus on promoting rational and equitable patient mobility. A key policy objective is to optimize regional medical resource allocation through the development of high-quality regional medical centers, thereby reducing unnecessary long-distance travel^16^. To support these efforts, it is essential to understand the driving forces behind intercity patient mobility. Identifying the roles of socioeconomic conditions, healthcare resource distribution, and geographic accessibility can inform more effective strategies to build a sustainable, tiered healthcare delivery system.

Previous research on intercity healthcare-seeking behavior has largely relied on linear models^16–18^, which assume uniform relationships between factors and outcomes across all locations. However, in reality, socioeconomic conditions, healthcare resource distribution, and geographic accessibility often interact in complex, non-linear ways, and their influence can vary greatly from one region to another. Few studies have systematically incorporated these spatial variations into a comprehensive framework, especially in the context of chronic diseases such as CKD. To address this, we employ a complementary mixed-method strategy. First, the Geodetector method is utilized to identify dominant correlates and quantify their globally non-linear impacts on patient mobility as well as the complex interactions between factors, without relying on strict linear assumptions. Second, the multiscale geographically weighted regression (MGWR) model complements this approach by allowing regression coefficients to vary across space, capturing spatial heterogeneity in the magnitude and direction of associations across cities^19^. By combining Geodetector’s capacity to detect global non-linear correlates with MGWR’s precision in characterizing locally heterogeneous associations, this study offers a coherent framework for disentangling the complex mechanisms shaping intercity patient mobility.

Therefore, this study employed a two-stage modeling framework integrating Geodetector and MGWR methods to examine the spatial correlates of intercity patient mobility and their spatial variation among CKD patients in China at the city level. Specifically, our research aims are: (1) utilizing the Geodetector model to systematically analyze and identify driving patterns of patient inflows and outflows; and (2) applying MGWR model to explore spatial variation in the associations of key factors. These insights will provide valuable evidence to inform policies on more equitable and effective allocation of nephrology resources across China, strengthening the healthcare system’s ability to respond to the rising burden of CKD.

## Results

### Geographic patterns of patient mobility

**Fig. 1:**
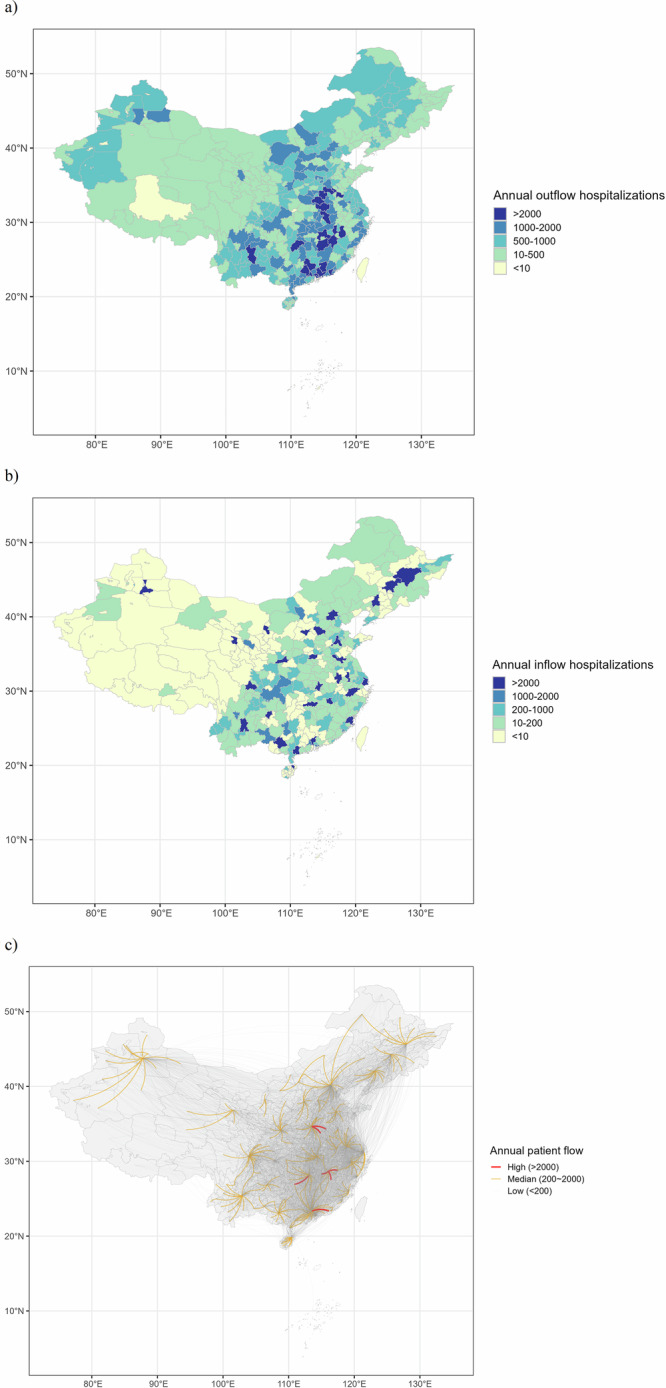
Spatial patterns of CKD-related intercity hospitalizations in China. The figure illustrates the geographical distribution and network connectivity of patient mobility at the city level. **a** Spatial distribution of annual outflow hospitalizations. **b** Spatial distribution of annual inflow hospitalizations. In **a**, **b**, colors represent the volume of hospitalizations: dark blue areas indicate the highest volume (>2000), transitioning through teal and green shades for intermediate volumes (10–2000), to yellow areas for the lowest volume (<10). **c** Network of annual inter-city patient flows. Lines connect origin and destination cities to visualize patient movement. Red lines represent high-volume flows (>2000 patients), orange lines represent median-volume flows (200–2000 patients), and gray lines represent low-volume flows (<200 patients). Map data source: National Geomatics Center of China.

Between 2014 and 2018, a total of 1332,189 CKD-related cross-city hospitalizations were recorded across 7441 directed city-level mobility paths in mainland China. The 360 source cities had an annual outflow of 740 (interquartile range [IQR] 576) hospitalizations, and the 250 destination cities had an annual inflow of 1066 (IQR 399) hospitalizations. As shown in Fig. 1a, cities with the highest patient outflows were mainly concentrated in central and southern China, with the top three being Zhoukou (average 3456 hospitalizations/year), Shangrao (average 3166 hospitalizations/year), and Shenzhen (average 3108 hospitalizations/year), suggesting limited local capacity for specialized CKD treatment in these regions. In contrast, Fig. 1b highlights that major inflow cities were mainly provincial capitals and megacities, with the top three being Guangzhou (average 31,932 hospitalizations/year), Zhengzhou (average 20,351 hospitalizations/year), and Changsha (average 15,257 hospitalizations/year), indicating their strong regional pull and concentration of advanced medical services.

The spatial distribution of the 13 potential driving factors used in subsequent analyses is illustrated in Fig. S2, revealing notable regional heterogeneity in economic, healthcare, and transportation characteristics across China.

### Identification of key factors using Geodetector

**Fig. 2:**
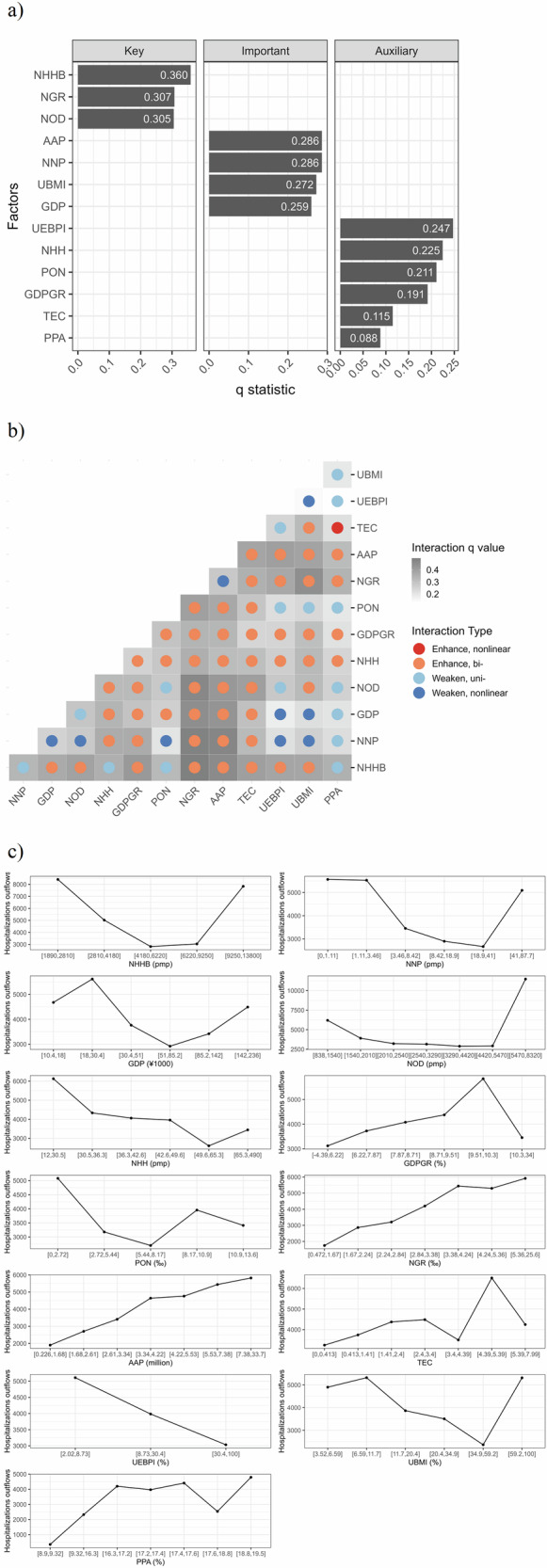
The Geodetector analysis results for CKD-related hospitalizations outflows. **a** Factor detector showing the q-statistic values representing the explanatory power of individual factors on spatial variation in CKD-related hospitalization inflows. **b** Interaction detector displaying the interaction associations between pairs of factors. **c** Risk detector showing the nonlinear associations between outflow volume and factors. AAP Average annual population, NGR Natural population growth rate, GDP City’s contribution to gross domestic product per capita, GDPGR GDP growth rate UEBPI Urban Employee Basic Pension Insurance coverage proportion, UBMI Urban Basic Medical Insurance coverage proportion, NHH Number of hospitals, NHHB Number of hospital beds, NOD Number of licensed doctors, NNP Number of nephrologists, PON Proportion of nephrologists per thousand doctors, TEC Traffic eigenvector centrality, PPA Proportion of the population aged 60 and over.

**Fig. 3:**
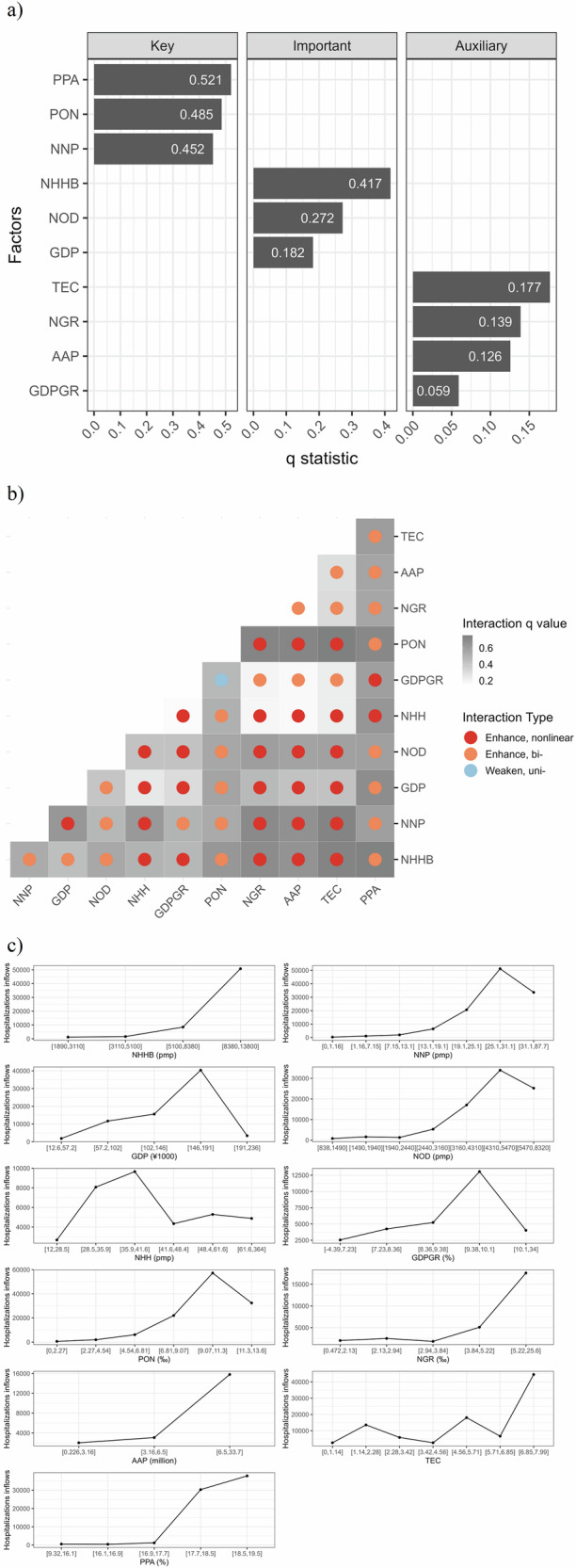
The Geodetector analysis results for CKD-related hospitalizations inflow. **a** Factor detector showing the q-statistic values representing the explanatory power of individual factors on spatial variation in CKD-related hospitalization inflows. **b** Interaction detector displaying the interaction associations between pairs of factors. **c** Risk detector showing the nonlinear associations between inflow volume and factors. AAP Average annual population, NGR Natural population growth rate, GDP City’s contribution to gross domestic product per capita, GDPGR GDP growth rate, NHH Number of hospitals, NHHB Number of hospital beds, NOD Number of licensed doctors, NNP Number of nephrologists, PON Proportion of nephrologists per thousand doctors, TEC Traffic eigenvector centrality, PPA Proportion of the population aged 60 and over.

**Fig. 4:**
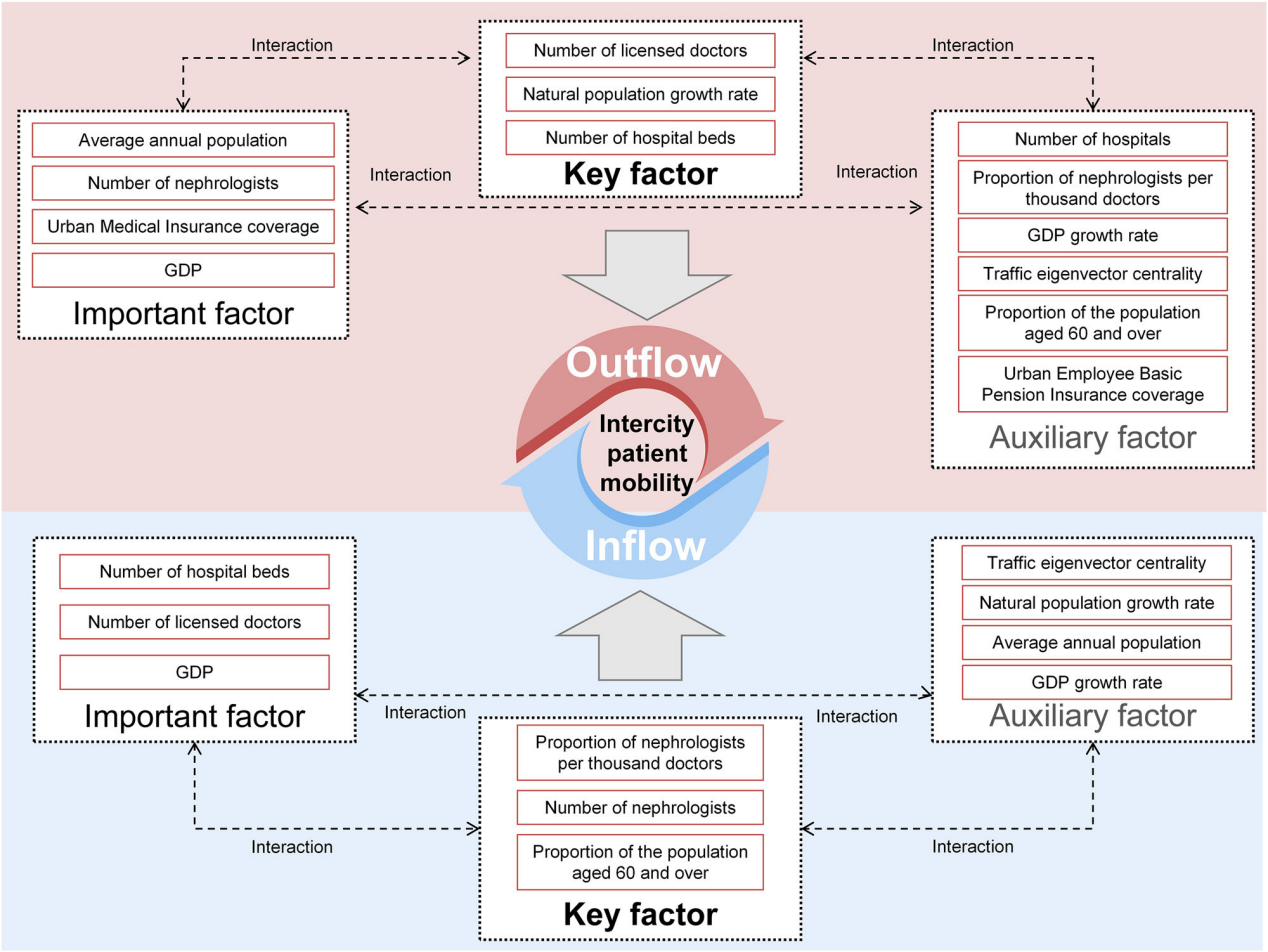
Driving mechanism of spatial heterogeneity in intercity patient mobility. GDP, City’s contribution to gross domestic product per capita.

Geodetector analysis was employed to systematically explore the spatial patterns and underlying correlates of CKD-related patient mobility across Chinese cities (Figs. 2 and 3). Based on findings of Geodetector analysis, we developed a framework for understanding the driving mechanism of intercity CKD patient mobility in China, as illustrated in Fig. 4.

For patient outflows, single-factor analysis (Fig. 2a) identified the top three factors as NHHB, NGR, and NOD, all classified as key factors. Specifically, NHHB accounted for 36.0% of the spatial variation in patient outflows (*q* = 0.360, *P* < 0.001), followed by NGR (*q* = 0.307, *P* < 0.001) and NOD (*q* = 0.305, *P* < 0.001). Additional important predictors included AAP, NNP, UBMI, and GDP, each explaining over 25% of the spatial variation. Interaction detection results (Fig. 2b) revealed widespread synergistic associations, with 52 factor pairs showing bilinear enhancement. Among these, combinations involving general medical resources and population factors exhibited the strongest explanatory power for CKD patient outflows. For example, the interactions of NHHB ∩ NGR (*q* = 0.496), NNP ∩ NGR (*q* = 0.490), and NNP ∩ AAP (*q* = 0.478) explained a substantial proportion of the spatial variation. Risk detector analysis (Fig. 2c) showed notable nonlinear associations. Specifically, both NHHB and NOD exhibited U-shaped relationships with patient outflows, indicating higher outflow volumes in cities with either very low or very high levels of these healthcare resources. In contrast, NGR showed a positively linear relationship, with higher outflows corresponding to higher rates of natural population growth.

For patient inflows, single-factor results (Fig. 3a) identified PPA (*q* = 0.521, *P* < 0.001), PON (*q* = 0.485, *P* < 0.001), and NNP (*q* = 0.452, *P* < 0.001) as the top three factors, each accounting for nearly half of the spatial variation in patient inflows. Additional important factors included NHHB, NOD, and GDP. Interaction detection results (Fig. 3b) identified 25 bilinear enhancements and 29 nonlinear enhancements among factor pairs, suggesting that most variables had synergistic associations when combined. Among these, combinations involving nephrology-specific medical resources and traffic connectivity factors exhibited the strongest explanatory power for CKD patient inflows. For example, the interactions of PON ∩ TEC (*q* = 0.753) and NNP ∩ TEC (*q* = 0.734) explained a substantial proportion of the spatial variation. Combinations involving general medical resources and population factors also exhibited a strong explanatory power for CKD patient inflows (NHHB ∩ PPA, *q* = 0.744). Risk detector analysis (Fig. 3c) revealed that patient inflows generally increase with higher levels of healthcare resource indicators (PON and NNP), indicating that cities with more hospital beds and higher nephrologist workforce availability tended to attract more patients. In the highest categories of both PON and NNP, a slight decline in patient inflows is observed. The analysis for PPA showed that inflows remained relatively stable at lower and moderate levels of aging but exhibited a non-linear surge in cities with high aging population proportion.

### Spatial heterogeneity of driving factors

To further investigate the spatially varying associations of key factors on the CKD-related patient mobility, MGWR models were constructed based on the top variables identified by the Geodetector factor detector.

**Fig. 5:**
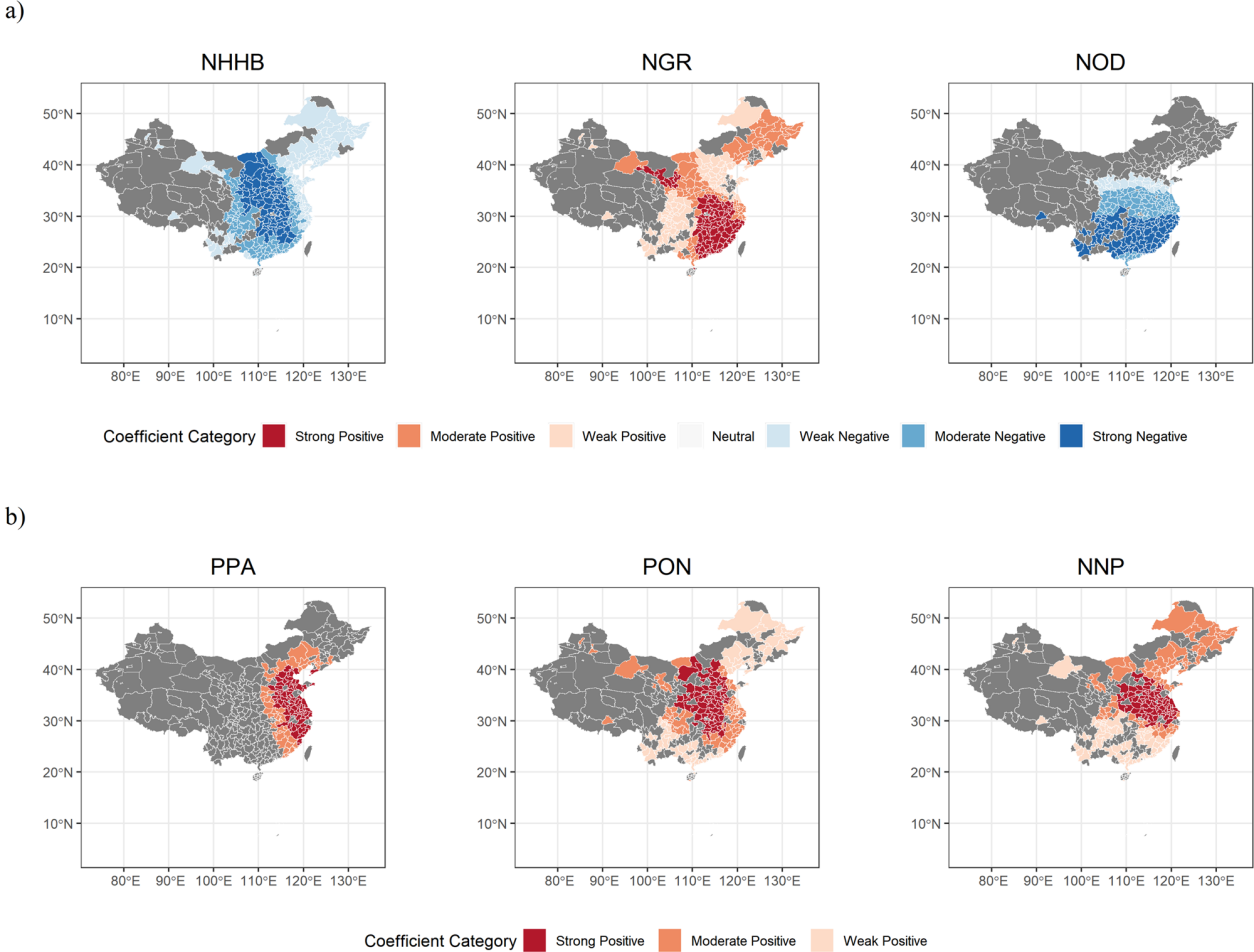
The spatial distribution of the local parameter estimates for the key influencing factors estimated using the MGWR model. **a** Outflow hospitalizations models showing spatial variation in the influence of number of hospital beds (NHHB), natural population growth rate (NGR), and number of licensed doctors (NOD). **b** Inflow hospitalizations models showing spatial variation in the influence of the proportion of the population aged 60 and over (PPA), proportion of nephrologists per thousand doctors (PON), and number of nephrologists (NNP). Only statistically significant local parameter estimates from the MGWR models are presented. Colors represent the direction and strength of the coefficients: Red shades indicate positive associations, categorized as dark red (Strong Positive), orange (Moderate Positive), and light peach (Weak Positive). Blue shades indicate negative associations, categorized as dark blue (Strong Negative), medium blue (Moderate Negative), and light blue (Weak Negative). Coefficients were classified into these six categories using tertile thresholds: Strong, Moderate, and Weak Negative for values below 0; and Weak, Moderate, and Strong Positive for values above 0. Gray areas represent regions with missing data. Map data source: National Geomatics Center of China.

For patient outflows (Fig. 5a), both NHHB and NOD in the source city showed strong negative associations with patient outflows. This suggests that cities with better healthcare capacity tended to retain more patients, reducing the need for them to seek care elsewhere. In contrast, a higher NGR was associated with greater patient outflows, particularly in the southeastern coastal areas.

For patient inflows (Fig. 5b), PPA exhibited a strong positive association, especially in the eastern coastal areas. Similarly, both PON and NNP in the destination city displayed broad clusters of positive associations across central and eastern China. These patterns suggest that in densely populated, competitive regions, cities with stronger nephrology workforce indicators are more likely to attract patients from other areas.

## Discussion

This study proposes a comprehensive framework to understand the spatial correlates of intercity CKD patient mobility in China and explores the geographic heterogeneity in their associations. By leveraging over 4 million hospitalization records and integrating Geodetector and MGWR methods, we reveal that disparities in both general and nephrology-specific medical resources are the primary factors associated with intercity patient mobility. Notably, there is substantial spatial heterogeneity and nonlinear interaction in the influence of socioeconomic and healthcare resource factors on intercity patient mobility. These findings underscore the need for region-specific strategies to enhance the efficiency and equity of CKD care delivery nationwide.

While previous research has emphasized the linear association between patient mobility and factors such as medical resources, socioeconomic conditions, and transportation^7,16,20^, few studies have discussed spatial heterogeneity and nonlinear interaction among these factors^19,21^. A prior study using the Geodetector model found that cross-provincial inpatient mobility in China was largely linked to medical resources, quality, and expenses, with their interactions accounting for regional inequalities in healthcare access^21^. Similarly, another study conducted in Henan province applying GWR models showed that local medical resources, health insurance coverage, and transportation accessibility were the most significant factors of county-level medical travel among cardiovascular and cerebrovascular disease patients in central China, while economic development and regional factors indirectly shaped these patterns^19^. Our study is the first to examine the nationwide pattern of patient mobility at the city level through the lens of geographic heterogeneity by integrating socioeconomic, transportation, and healthcare resource factors into a comprehensive framework. We further explore the nonlinear and interactive associations of these multiple correlates and use MGWR to analyze how these complex associations vary across different regions.

Our analysis indicates a distinct differentiation between outflow and inflow correlates. Specifically, higher patient outflows are primarily associated with lower availability of general medical resources, such as doctor and bed density of source cities, while patient inflows are more strongly linked to the nephrology-specific medical resources in destination cities. The two-way interaction analysis revealed that spatial variations in CKD patient mobility were not associated with single factors alone but with the nonlinear interplay of multiple influences. Among these, the combination of healthcare resources and population size had the strongest explanatory power for patient outflow. This suggests that when demographic pressures coincide with limited healthcare capacity, patients are more likely to seek treatment outside their home city. These results extend prior research using generalized linear models showing that patient outflow was significantly associated with healthcare capacity and city population size (*P* < 0.01)^20^. The most influential interactive factors on the spatial variation of CKD patient inflow were the combinations involving nephrology-specific medical resources and traffic connectivity factors. In cities with more nephrology-specific medical resources, strong traffic connectivity may amplify the city’s role as a healthcare hub by lowering geographic barriers for incoming patients.

In non-linear analysis, we identified U-shaped relationships between both the NHHB and NOD with patient outflows, aligning with prior research findings^7^. In resource-limited cities, higher outflows likely reflect insufficient local capacity, whereas in well-resourced cities, increased outflows may arise from both individual and system-level factors. At the individual level, improved health awareness, stronger treatment intentions, and greater economic ability may enable patients to seek care elsewhere. At the system level, referral spillovers to higher-level hospitals, medical tourism for specialized services, administrative boundary effects, and differences in hospital specialization or quality not captured by resource counts may also contribute. For patient inflows, we observed that the attractiveness of cities tends to increase with nephrology workforce availability, but may plateau or even decline beyond a certain threshold. These nonlinear trends suggest that there is an optimal range for both the absolute number and density of nephrologists. Prior evidence proposes a range of 12–20 nephrologists per million population as optimal based on local needs^7^. The positive association between the aging population proportion (PPA) and patient inflows suggests that cities with higher PPA generally possess stronger economic foundations and better medical service levels, which enhance their attractiveness to patients. In China, areas with higher aging ratios are often economically developed regions with well-established healthcare systems, reflecting long-term resource accumulation and service accessibility that draw patients from surrounding areas. However, we acknowledge that this relationship likely involves reverse causality. As highlighted in national policy analyses, the spatial distribution of healthcare capacity is also responsive to disease burden. Regions with historically high patient demand have been prioritized in national strategies for infrastructure expansion and specialty development to bridge the gap between supply and demand^22^. This creates a reinforcing feedback loop: high capacity attracts patients, and the resulting concentration of complex cases further justifies government investment and resource allocation. Further prospective studies are warranted to better elucidate the causal relationships between healthcare resources and patient mobility.

This study further advances existing research by capturing significant spatial heterogeneity in the correlates of patient mobility. The MGWR results indicate complex spatial patterns in the relationships between medical resource indicators and patient outflows. In most regions, higher numbers of hospital beds and doctors were associated with reduced patient outflows, reflecting the expected association of improved local service capacity. For inflows, the northern regions were more influenced by the absolute numbers of nephrologists and beds, indicating that capacity remains a critical factor. In contrast, in the southeastern regions, patient inflows were more closely associated with nephrologists, suggesting that healthcare efficiency and specialization may play a greater role in areas with relatively abundant healthcare resources. As key variables were highly correlated and therefore examined in separate MGWR models, the estimated spatial effects should be interpreted as independent associations rather than mutually adjusted effects. These spatial variations underscore the importance of tailoring health policies that account for local socioeconomic conditions, healthcare demand, and existing patterns of medical resource distribution.

The observed patterns of CKD patient mobility have important implications for health system design, particularly regarding the efficiency of healthcare resource use and the spatial equity of access. From an efficiency perspective, patient mobility may increase utilization of underused high-level facilities and improve outcomes where service capacity is highly uneven. However, rising patient flows toward tertiary centers can also increase costs, worsen congestion, and strain specialized resources, potentially undermining overall system performance. Optimizing patient mobility is therefore essential to achieving cost-effective service delivery. Strategies that strengthen regional coordination, reinforce tiered delivery systems, and refine reimbursement and referral policies may help align mobility with desired system goals. First, our findings highlight the joint influence of healthcare resource distribution and transportation connectivity on patient mobility. Integrated planning between health system development and transport infrastructure is needed to support coordinated regional referral networks that reduce unnecessary long-distance travel while preserving access to high-quality specialized care. Such governance mechanisms can help ensure that mobility contributes to, rather than fragments, system efficiency. Second, spatial heterogeneity in the driving patterns of mobility should be considered when strengthening tiered delivery arrangements. For example, in central China, enhancing the capacity of primary care may improve system efficiency because mobility is more strongly linked to general healthcare capacity. In contrast, in eastern China, socioeconomic factors play a greater role; thus, promoting a more balanced distribution of both primary and specialized services may be more effective in meeting increasing healthcare needs. Third, the observed U-shaped relationship between medical insurance coverage and mobility suggests that insurance design may have unintended consequences for patient mobility. In regions with relatively low coverage, expanding insurance may reduce outward mobility by enabling greater use of local services. Conversely, in regions with already high coverage, increased patient mobility may reflect unintended effects of reimbursement and referral policies; in such contexts, improving resource allocation and access to high-quality services may be more important levers for managing mobility.

With respect to equity in healthcare access, patient mobility and healthcare equity are mutually constitutive. Mobility is not only linked to unequal distributions of healthcare resources but may also further undermine equity in both source and destination regions. In source cities, outward mobility may exacerbate inequities by widening gaps between smaller and larger cities and by amplifying differences in care-seeking opportunities between socioeconomically advantaged and disadvantaged groups. In destination cities, large inflows of nonlocal patients may compromise the availability and quality of services for local residents. Equitable healthcare development requires not only expanding overall capacity but also managing its spatial distribution to ensure proportional access across regions. Developing flexible allocation guidelines, such as regional benchmarks for nephrologist density, can help prevent both under-provision and over-concentration, ensuring that investments in healthcare resources translate into meaningful improvements in patient access and system efficiency.

Certain limitations of this study should be acknowledged. First, our analysis was restricted to patient mobility data from tertiary hospitals and city-level flows with more than five hospitalizations during the study period, which may have introduced selection bias. However, given that cross-city hospitalization is most commonly sought in tertiary hospitals, the data likely remain broadly representative of CKD-related mobility. Second, the use of aggregated city-level data may result in ecological fallacy, whereby group-level associations are inaccurately attributed to individuals. As such, caution is warranted when interpreting the findings at the individual level. Third, the nephrology workforce may be underestimated, as data sourced from online healthcare platforms could underrepresent providers in less urbanized areas or regions with lower hospital prestige and patient demand. Fourth, although we included the number and proportion of nephrologists as indirect proxies, the lack of direct indicators of healthcare quality, such as hospital performance or treatment outcomes, may have introduced bias, as these factors could partially mediate patient mobility decisions. Fifth, we acknowledge that the omission of other potential sociodemographic determinants (e.g., education level and income inequality) due to data unavailability may limit the comprehensiveness of our analysis. Sixth, although global nonlinearity was observed in the associations between some factors (such as NHHB and NOD) and patient mobility, the MGWR captures spatial heterogeneity in locally linear associations but cannot model spatially varying non-linear relationships. Future studies could extend this analysis by incorporating non-linear spatial models or machine learning approaches to further explore complex spatial dependencies. Seventh, considering potential multicollinearity and to simplify the assessment of heterogeneity in total effects, interaction terms were not included in the MGWR model, which may introduce some estimation bias. However, sensitivity analysis incorporating the top interaction terms identified by Geodetector yielded similar spatial patterns (Fig. S3), albeit with greater uncertainty in the coefficient estimates. Eighth, running separate MGWRs for different focal variables may obscure potential joint or interactive effects among covariates, and thus raises concerns about the comparability of spatial patterns and effect magnitudes across models. However, sensitivity analysis incorporating all factors yielded similar spatial patterns (Fig. S5), though accompanied by greater uncertainty in the coefficient estimates. Additionally, our analysis was based on cross-sectional data from a specific study period, which limits our ability to assess changes in patient mobility patterns or healthcare resource distribution over time. The cross-sectional design also limits causal inference, as reverse causality between healthcare resources and patient flow cannot be ruled out. Future studies using longitudinal data could capture temporal dynamics and policy impacts more effectively. Finally, while this study focused on node-based characteristics to evaluate both the spatially heterogeneous and nonlinear factors influencing patient mobility, future research could employ gravity or spatial interaction models to examine the bidirectional connectivity and flow magnitudes between specific origin-destination pairs.

This study enhances understanding of intercity mobility for CKD patients by integrating spatial modeling with real-world, city-level health resource data. These findings underscore the need for differentiated healthcare policies that optimize regional medical resource allocation based on empirical evidence of patient mobility patterns and healthcare accessibility. Future research should extend this analytical framework to other chronic conditions and explore the dynamic relationship between healthcare resource investment and patient mobility patterns to inform evidence-based policy development.

## Methods

### Study area

China, located in East Asia, spans approximately 9.6 million square kilometers and has a population exceeding 1.4 billion. The country is administratively organized into three levels: provincial, city, and township. Among its 34 provincial-level divisions, 31 are located in mainland China. Each province encompasses multiple prefecture-level cities, which serve as key administrative and economic units and form the basis for spatial analyses in this study. Significant geographic disparities exist in the distribution of healthcare resources across Chinese cities. Medical resources, such as advanced medical expertise and specialized services, are often concentrated in provincial capitals and economically developed megacities. As a result, patients frequently engage in cross-city mobility to seek better healthcare services when local provisions are insufficient in capacity or quality. Prominent urban centers such as Beijing, Shanghai, and Guangzhou function as national healthcare hubs, attracting patients from distant provinces who are willing to travel long distances to access superior healthcare services^23^.

### Patient mobility data

City-level patient mobility data were derived from the Hospital Quality Monitoring System (HQMS), a mandatory, nationwide database maintained by the National Health Commission of the People’s Republic of China. The HQMS is primarily used for hospital accreditation and collects standardized electronic discharge records from the front page of inpatient medical records (MRFP) in tertiary hospitals. Tertiary hospitals, defined as top-level institutions with at least 500 beds, serve as the highest tier in China’s healthcare system, capable of delivering primary, secondary, and specialized tertiary care. These hospitals play a central role in cross-regional patient care, as they accommodate a large share of the national inpatient population; in 2019, approximately 54% of all hospital inpatients in China were treated in tertiary hospitals. The MRFP includes 346 structured patient-level variables encompassing demographic characteristics, diagnoses, and procedures, with all personally identifiable data removed. Completed by attending doctors as part of standard legal protocol, the MRFP ensures the accuracy and completeness of medical information. Additionally, the HQMS system employs automated quality control checks at the time of data submission to ensure consistency and reliability. As of December 2018, the database covered over 75% of tertiary hospitals across 31 provinces and 359 cities in mainland China, excluding Hong Kong, Macao, and Taiwan. More detailed descriptions of these data sources can be found in existing literature^24,25^.

In this study, intercity patient mobility was measured using CKD-related cross-city hospitalizations. We extracted hospitalization records from January 2014 to December 2018 for patients diagnosed with CKD, as defined by International Classification of Diseases, 10th Revision (ICD-10) codes, including diabetic nephropathy, hypertensive nephropathy, glomerulonephritis, tubulointerstitial nephritis, obstructive nephropathy, and related conditions (Table S1). CKD-related hospitalization was identified when the disease appeared as either the primary or one of the first two secondary diagnoses. Multiple hospitalization records per patient were retained. Cross-city hospitalization was identified by comparing the patient’s residential location (source city) with the city of hospitalization (destination city). Source cities were determined using a combination of residential address, postal code, and contact number information recorded in the MRFP. From 6,415,559 CKD-related hospitalizations, 1,367,471 (21.3%) were identified as cross-city, spanning 26,082 unique directed city-level mobility paths. To ensure data representativeness and avoid sparse data bias, we included only those city-level mobility paths with more than five hospitalization records during the study period. A total of 1,332,189 cross-city CKD-related hospitalizations were included, comprising 7441 directed city-to-city mobility paths. These aggregated inflow and outflow counts of CKD-related hospitalizations at the city level served as the primary outcome variables for subsequent statistical analyses (Fig. 1). This study involved human participants and was approved by the Ethics Committee of Peking University First Hospital (2020-018). The study was conducted in accordance with the Declaration of Helsinki. The requirement for informed consent was waived by the Ethics Committee of Peking University First Hospital due to the use of de-identified administrative data.

### Socioeconomic data

To examine the potential correlates of CKD patient mobility, we compiled a multisource dataset characterizing the socioeconomic, healthcare, and transportation attributes of Chinese cities. A total of 13 indicators were selected based on existing literature^7,26,27^ to capture regional disparities relevant to patient mobility. Specifically, annual population growth rate (NGR) and GDP growth rate (GDPGR) were included to reflect the “push-pull” effects of socioeconomic dynamics on patient mobility, as rapid population growth may increase healthcare demand pressure, while economic growth often enhances healthcare infrastructure and service attractiveness. A complete list of indicator definitions and data sources is provided in Table S2. For this study, the term “city” refers to the administrative area governed by a prefecture-level municipality.

Sociodemographic indicators included average annual population (AAP), natural population growth rate (NGR), and proportion of the population aged 60 and over (PPA). AAP and NGR were obtained from LandScan, a high-resolution global population dataset that integrates demographic, geographic, and remote sensing data to model resident population distribution^28^. PPA was sourced from the China Statistical Yearbook^29^. Economic indicators, including gross domestic product (GDP) per capita and GDP growth rate (GDPGR), were sourced from the China City Statistical Yearbook^30^. The Urban Employee Basic Pension Insurance (UEBPI) and the Urban Basic Medical Insurance (UBMI) coverage proportion during the study period (2014–2018) were also sourced from the China City Statistical Yearbook^30^.

Healthcare resource indicators included the number of hospitals (NHH), hospital beds (NHHB), licensed doctors (NOD), nephrologists (NNP) per million population, and the proportion of nephrologists per thousand doctors (PON). NHH, NHHB, and NOD were sourced from the China City Statistical Yearbook^30^. The precise size and geographic distribution of the nephrology workforce in mainland China remain unclear due to limited dedicated studies and the absence of a centralized registration system^7^. Although a joint questionnaire survey by ASN, ERA-EDTA, and ISN reported the total number of licensed nephrologists in China^31^, these data do not capture actively practicing, patient-accessible specialists or provide geographic distribution at the city level. To better estimate the availability of specialized kidney care, we used the number of nephrologists registered on two major Chinese online medical consultation platforms (Haodf.com and WeDoctor.com) as a proxy for high-quality, well-recognized specialists. These data serve as a reliable proxy for the effective and accessible physical workforce, given the extensive coverage of the platforms, consistency with national workforce estimates reported in the literature, and manual validation of data accuracy^25,32,33^. The detailed methodology for data extraction and validation of nephrology workforce can be found in our previous study^7^ and supplementary Text S2. PON was calculated as the ratio of NNP to NOD, providing an indicator of the specialization level within the local medical workforce.

Transportation accessibility was captured using traffic eigenvector centrality (TEC), calculated from an intercity transportation network comprising flight and train connections. In this network, cities were treated as nodes, and direct transportation routes (air or rail) as edges. Transportation data were sourced from OpenFlights and OpenStreetMap dataset. Eigenvector centrality measures a city’s relative importance within the network based on both the quantity and quality of its connections, thus reflecting the ease with which patients can travel to or from a city for healthcare purposes (see Supplementary Text S1 for methodological details)^34^.

### Statistical analysis

**Fig. 6:**
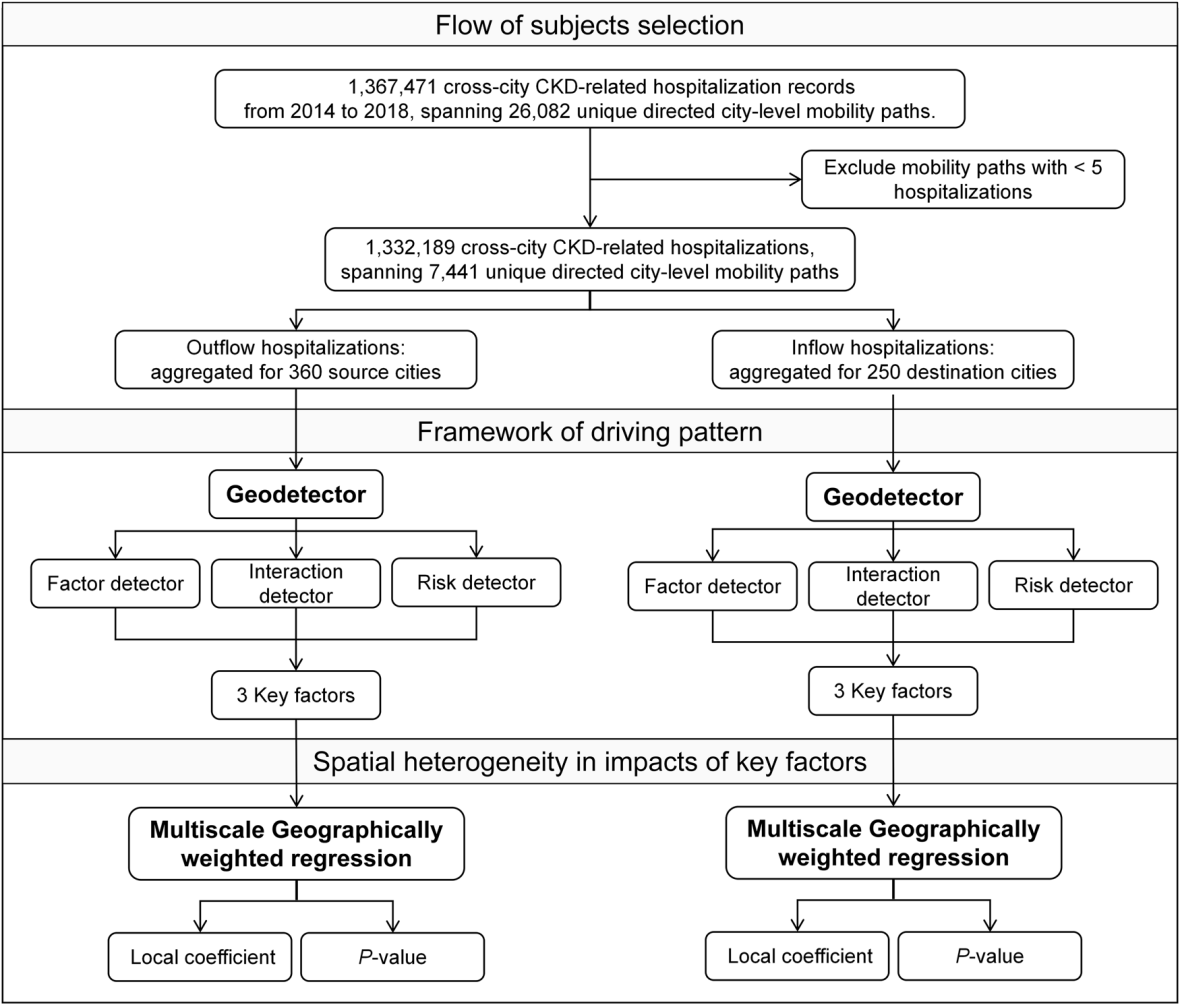
Framework for the study design. The flowchart outlines the three-stage methodological approach employed in this study. Top panel: The “Flow of subject selection” illustrates the data processing criteria. Middle panel: The “Framework of driving pattern” details the use of the Geodetector model, comprising factor, interaction, and risk detectors, to screen and identify the top three key driving factors for patient mobility. Bottom panel: The “Spatial heterogeneity in impacts of key factors” describes the subsequent application of Multiscale Geographically Weighted Regression (MGWR) to calculate local coefficients and *P*-values, examining the spatially varying impacts of the identified factors. CKD Chronic Kidney Disease.

Statistical analyses in this study would be conducted according to the framework shown in Fig. 6. We first described the overall scale and geographic distribution of CKD-related patient mobility across mainland China, alongside descriptive statistics of potential explanatory variables.

To identify cross-city patient mobility patterns, we applied the Geodetector model separately for CKD patient outflow and inflow, a spatial statistical method designed to assess the explanatory power of candidate variables in spatial variation. The model quantifies the extent to which a factor *X* explains the spatial variation of a dependent variable *Y*, and it can also evaluate the interaction associations between multiple factors^35,36^. In traditional Geodetector, the discretization of spatial data is heavily influenced by subjective judgment. This study employed an optimal-parameter-based geographical detector model, which can automatically select discretization methods and determine intervals for continuous variables. It explored the optimal combination of spatial scale, spatial data discretization methods, and the number of spatial intervals to achieve more precise spatial analysis, thereby improving the accuracy and effectiveness of the model^37,38^. In this study, *Y* represents the observed number of CKD-related hospitalizations inflow or outflow for each city. Three components of the Geodetector model were used in this study: (1) The factor detector was applied for single-factor analysis, using a statistic *q*∈[0,1] to measure the degree of influence of a certain factor *X* on dependent variable *Y*^39^. The *q*-statistic is computed as:

1 $${\rm{q}}=1-\frac{1}{{N}_{{\sigma }^{2}}}{\sum }_{h=1}^{L}{N}_{h}{\sigma }_{h}^{2}$$

where *N* and *σ*^*2*^ represent the total sample size and variance of *Y* in the entire study area; *h* = 1, 2, 3, …, L, where *L* is the number of strata (sub-regions or sub-classes) of factor *X*; *N*_*h*_ and *σ*^*2*^_*h*_ are the sample size and variance of *Y* in stratum ℎ, respectively. By evaluating the *q*-statistic, we identified the most influential factors associated with variations in CKD patient inflow and outflow across regions, respectively. (2) The interaction detection is used to evaluate whether the combination of two factors *X*_*1*_ and *X*_*2*_ enhances or weakens their joint explanatory power on *Y*. This is done by comparing *q*(*X*_*1*_), *q*(*X*_*2*_) and *q*(*X*_*1*_ ∩ *X*_*2*_), resulting in five possible interaction types: weakened, independent, bilinear enhancement, or nonlinear enhancement or suppression (Fig. S1). (3) The risk detector was employed to examine potential nonlinear associations of driving factors by identifying statistically significant differences in the mean values of the dependent variable *Y* across different strata (i.e., categories or intervals) of an independent variable *X*, based on a two-sample *t*-test. To visualize these associations, we plotted the mean risk, defined as the average CKD-related hospitalizations inflow or outflow, for each stratum of the explanatory variables using line charts.

Utilizing the output of the Geodetector model, we established a framework for understanding the driving patterns of intercity CKD patient mobility in China. First, we applied the factor detector to quantify the explanatory power (*q*-value) of each potential influencing factor, enabling the identification of key factors (highest *q* values) and important factors (moderately high *q* values) that shape patient flows. Although MGWR coefficients can reflect the local strength of relationships, we used Geodetector to assess the overall importance of factors because it allows for evaluation of all variables without the multicollinearity limitations inherent in MGWR. Second, we employed the interaction detector to examine the joint impact of pairs of factors on mobility patterns, allowing for a systematic understanding of both the individual and interactive roles of multiple factors in CKD intercity patient mobility.

To further explore the spatial heterogeneity in the impact of key factors, we constructed six MGWR models, three for patient outflow and three for patient inflow. Each model used one of the top three Geodetector-identified variables as the main explanatory variable, with additional covariates included based on model fit. Key variables were modeled separately due to their high intercorrelation, which led to elevated variance inflation factors (VIF) and potential instability in coefficient estimates when included simultaneously. Interaction terms were not considered in the MGWR models to simplify the estimation of total effects and avoid potential multicollinearity. Based on Sachdeva’s theory^40^, we generated diagnostic plots of local parameter estimates (*β*) from the MGWR models against the corresponding scaled values of key variables that exhibited significant global non-linear associations in the Geodetector analysis (NHHB and NOD) to assess whether spatial heterogeneity was present for key variables (see more details in Supplementary Text S3). The diagnostic plots (Fig. S4) showed no discernible structure, supporting the presence of spatially varying processes. Unlike traditional global regression models that assume spatial stationarity, MGWR allows the model coefficients to vary across geographic space, thereby capturing localized relationships between predictors and patient mobility outcomes. MGWR introduces geographic coordinates into the estimation process and applies a non-parametric, locally weighted regression approach to estimate location-specific parameters. The MGWR model can be expressed as:

2 $${y}_{i}={\beta }_{0}({u}_{i},{v}_{i})+\mathop{\sum }\limits_{k=1}^{i}{\beta }_{k}({u}_{i},{v}_{i}){x}_{ik}+{\varepsilon }_{i}$$

where *y*_*i*_ is the observed value of CKD-related hospitalizations inflow or outflow for city *i*; (*u*_*i*_, *v*_*i*_) is the spatial coordinates (centroid) of city *i*, *β*_*0*_(*u*_*i*_, *v*_*i*_) is the city-specific intercept, *β*_*k*_(*u*_*i*_, *v*_*i*_) is the city-specific regression coefficient for the *k*-th explanatory variable, *x*_*ik*_ is the value of the *k*-th explanatory variable for city *i*, and *ε*_*i*_ is the residual term, assumed to be normally distributed with mean zero. Covariate-specific adaptive bandwidths were determined using the cross-validation method to capture the distinct spatial scale of influence for each variable. The use of adaptive bandwidths is particularly appropriate for city-level units with heterogeneous spatial density, as in China. Under this framework, cities located in densely populated regions are calibrated using smaller bandwidths, while cities in sparsely distributed regions rely on larger bandwidths to ensure stable local estimation based on a comparable number of neighboring observations. Prior to modeling, multicollinearity among other variables was assessed using the VIF, and only variables with VIF values less than 5 were retained for inclusion in the models, as detailed in Supplementary Table S3.

All statistical tests were two-sided, with *P* < 0.05 considered statistically significant. Analyses were conducted using R software (version 4.4.0; R Foundation for Statistical Computing, Vienna, Austria). The *GD* package was used to implement the Geodetector model, and the *GW model* package was used for MGWR modeling.

## Supplementary information

Supplementary Material.

## Data Availability

The datasets generated and analyzed during the current study are not publicly available due to privacy and ethical restrictions, but are available from the corresponding author, Pengfei Li, on reasonable request.
