# Supplementary material for: Spatially heterogeneous and nonlinear factors influencing intercity patient mobility for chronic kidney disease: a nationwide study in China
Source: Npj Health Syst. 2026 May 18;3:32. doi: 10.1038/s44401-026-00086-z (PMC13336799; doi:10.1038/s44401-026-00086-z)
Supplement: Supplementary file 1 — Supplementary Material. [file 44401_2026_86_MOESM1_ESM.docx]

**Supplementary Material**

**Title: Spatially Heterogeneous and Nonlinear Factors Influencing Intercity** **Patient Mobility for Chronic Kidney Disease: A Nationwide Study in China**

**Text S1. Traffic eigenvector centrality**

Eigenvector centrality was originally developed within graph theory as a metric for quantifying vertex influence within network structures. This concept has gained widespread adoption across diverse research domains, and traffic eigenvector centrality has been a commonly-used measure of traffic development in recent studies^1 2^. The traffic eigenvector centrality evaluates the centrality of a vertex (i.e., city) in the transportation network using the weighted sum of centralities from neighboring vertices. For a vertex *v*, the traffic eigenvector centrality *x_v_* could be calculated using a power iteration strategy as follows.

$$x_{v}=\frac{1}{\lambda}\sum_{t\in M(v)} x_{t}=\frac{1}{\lambda}\sum_{t\in V} a_{v,t}x_{t}$$

For a given transportation network *G* = (*V*, *E*), where *V* denotes the vertices and *E* denotes the edges. The adjacency matrix *A* = (*a_v_*_,_*_t_*) defines the network structure: *a_v_*_,_*_t_* equals 1 when vertex *v* connects to vertex *t*, and equals 0 in the absence of such connection. *M*(*v*) denotes the neighbor set for vertex *v*, while *λ* represents a constant parameter.

**References**

1. Reza S, Ferreira MC, Machado JJM, et al. Road networks structure analysis: A preliminary network science-based approach. *Annals of Mathematics and Artificial Intelligence* 2022 doi: 10.1007/s10472-022-09818-x

2. Azimifar Z, Soltani sarvestani M, Safavi AA, et al. Expanding the eigenvector centrality for multi-layer graphs and its application in managing traffic and urban infrastructure. *Quarterly Journal of Transportation Engineering* 2020:-. doi: 10.22119/jte.2020.119374

**Text S2. Validity and Representativeness of Online Nephrology Workforce Data**

To assess the nephrology workforce capacity in China, this study utilized data aggregated from two major online healthcare platforms, Haodaifu and WeDoctor. While these are digital platforms, the data extracted serves as a reliable proxy for the effective and accessible physical workforce. This reliability is supported by the extensive coverage of the platforms, consistency with existing literature, and manual validation of data accuracy.

Firstly, the data sources for this study, Haodaifu and WeDoctor, are the leading healthcare platforms in China with massive coverage. Haodaifu aggregates information on over 890,000 physicians from more than 10,000 hospitals, while WeDoctor covers 240,000 physicians across more than 7,200 hospitals. Crucially, these platforms do not rely solely on voluntary physician sign-ups; they actively amass extensive data directly from official hospital websites, encompassing details such as location, seniority, specialty, and hospital affiliation.

Secondly, the workforce estimates derived from this real-world data align with and refine previous estimates found in the literature. The workforce estimates identified a total of 12,894 nephrologists, resulting in a density of 9.13 nephrologists per million population (pmp). This density is highly consistent with the global median of 9.1 nephrologists pmp reported in a joint investigation by the American Society of Nephrology (ASN), European Renal Association-European Dialysis and Transplant Association (ERA-EDTA), and International Society of Nephrology (ISN). While a previous report estimated China’s nephrology workforce at approximately 8,500 (6.2 pmp), our study utilizes granular, real-time data to provide a more precise count. The alignment with global medians suggests that the platform data does not grossly over- or underestimate the workforce but rather provides a comprehensive, updated census.

Finally, to verify that the online data reflects the actual physical staffing of hospitals (effective capacity) rather than just "digital presence", this study conducted a manual validation process by comparing the platform data against the actual staffing records of 15 randomly selected hospitals: 10 urban hospitals located in developed cities (Beijing, Shanghai, Guangzhou, and Shenzhen) and 5 hospitals in rural regions. The results demonstrated high data fidelity. The mean absolute error (MAE) between the platform data and the actual hospital counts was only 2 nephrologists for urban hospitals and 1 nephrologist for rural hospitals. This negligible margin of error confirms that the web-crawled data accurately mirrors the real-world distribution of nephrologists. Therefore, the dataset is a valid proxy for the accessible nephrology workforce capacity available to patients in these regions.

References

Wu J, Li Q, Bao C, Yang C, Li P, Zhang L. Nephrology workforce in China: describing current status and evaluating the optimal capacity based on real-world data. Human Resources for Health. 2023;21(1):62.

**Text S3.** **Diagnostic Testing for Non-linearity and Spatial Heterogeneity**

Following the framework of Sachdeva et al. (2022), we conducted a diagnostic screening to determine whether the observed spatial variations in the local parameter estimates from our GWR model were truly representative of spatially varying processes or were manifestations of misspecified global non-linear relationships.

As established in the literature, if the relationship between a covariate and the dependent variable is non-linear and the covariate exhibits spatial dependency, a local model may produce spatially varying parameter estimates that mirror the spatial pattern of the covariate itself. To differentiate these mechanisms for NHHB and NOD, variables with high non-linear associations identified by Geodetector, we plotted the local parameter estimates (*β*) against their respective covariate values (NHHB and NOD). Rather than a totally random scatter, a non-random systematic trend in such a plot indicates that the parameter estimates are a function of the covariate, confirming an underlying non-linear relationship. Conversely, the absence of discernible structure would support the claim of pure spatial non-stationarity.

Our diagnostic results for NHHB and NOD demonstrate a non-random systematic trend (**Figure S4**), confirming that non-linearity is a contributing factor to parameter variation. However, the geographical uniqueness of these variables is confirmed by the significant "spatial mismatch" between the distribution of the covariates (**Figure S1**) and the highly clustered regional patterns of the GWR coefficients (**Figure 6**). This indicates that while non-linearity exists, it does not fully account for the regional sensitivity and spatial heterogeneity captured by the local model.

**References**

Sachdeva M, Fotheringham AS, Li Z, Yu H. Are We Modelling Spatially Varying Processes or Non-linear Relationships? Geographical Analysis. 2022;54(4):715-38

**Table S1. The International Classification of Diseases-10 coding of CKD.**

| Etiology of CKD | All editions | China edition | Beijing edition | Clinic edition |
| --- | --- | --- | --- | --- |
| 1. Diabetes mellitus |  |  |  |  |
| Type 1 diabetes mellitus with renal complications | E10.2+ N08.3 |  |  |  |
| Type 2 diabetes mellitus with renal complications | E11.2+ N08.3 |  |  |  |
| Unspecified diabetes mellitus with renal complications | E14.2 |  |  |  |
| Malnutrition-related diabetes mellitus with renal complications |  | E12.200+N08.3 |  | E12.200 |
| Other specified diabetes mellitus with renal complications |  | E13.2 |  | E13.200 |
| 2. Hypertensive diseases |  |  |  |  |
| Hypertensive renal disease with renal failure | I12 |  |  |  |
| Hypertensive heart and renal disease with (congestive) heart failure | I13 |  |  |  |
| Pregnancy with hypertensive heart and renal disease | O10.301 |  |  |  |
| Pregnancy with essential hypertension and proteinuria | O11.x01 |  |  |  |
| Pre-existing hypertensive renal disease during pregnancy,  childbirth and puerperium |  | O10.200 |  | O10.200 |
| Pregnancy with hypertensive renal disease |  | O10.201 |  | O10.201 |
| Pre-existing hypertensive heart and renal disease during pregnancy, childbirth and puerperium |  | O10.300 |  | O10.300 |
| Pre-existing hypertension with proteinuria |  | O11.x00 |  | O11.x00 |
| 3. Glomerular diseases |  |  |  |  |
| Recurrent and persistent hematuria | N02 |  |  |  |
| Chronic nephritic syndrome | N03 |  |  |  |
| Nephrotic syndrome | N04 |  |  |  |
| Unspecified nephritic syndrome | N05 |  |  |  |
| Isolated proteinuria with specified morphological lesion | N06 |  |  |  |
| Persistent proteinuria, unspecified | N39.1 |  |  |  |
| 4. Renal tubulointerstitial diseases |  |  |  |  |
| Chronic tubulointerstitial nephritis | N11 |  |  |  |
| Tubulointerstitial nephritis, not specified as acute or chronic | N12 |  |  |  |
| Drug- and heavy-metal-induced tubulointerstitial and tubular conditions | N14 |  |  |  |
| Renal tubulointerstitial disorders in diseases classified elsewhere | N16 |  |  |  |
| Other specified disorders of carbohydrate metabolism | E74.8 |  |  |  |
| Disorders of amino-acid transport | E72.0 |  |  |  |
| Nephrogenic diabetes insipidus |  | N25.1 |  | N25.1 |
| Renal tubule acidosis | N25.8 |  |  |  |
| Balkan nephropathy |  | N15.000 | N15.001 | N15.000 |
| Renal tubulointerstitial disease, specified |  | N15.800 |  | N15.800 |
| Renal granuloma |  | N15.801 |  | N15.801 |
| Renal tubulointerstitial disease |  | N15.900 |  | N15.900 |
| Impaired renal tubular function-related disease |  | N25.9 |  | N25.9 |
| Liddle syndrome |  | I15.101 |  | I15.101 |
| Urate nephropathy |  | M10.001+N16.8 | N28.905 | M10.001+N16.8 |
| Systemic lupus erythematosus + renal tubulointerstitial diseases |  | M32.102+N16.4 | M32.113+N16.4 | M32.102+N16.4 |
| Sicca syndrome + renal tubulointerstitial diseases |  | M35.006+N16.4 | M35.005+N16.4 | M35.006+N16.4 |
| 5. Obstructive nephropathy |  |  |  |  |
| Hydronephrosis with ureteropelvic junction obstruction | N13.0 |  |  |  |
| Hydronephrosis with ureteral stricture, not elsewhere classified | N13.1 |  |  |  |
| Hydronephrosis with renal and ureteral calculous obstruction |  | N13.2 | N13.2 | N13.200 |
| Other obstructive nephropathy |  | N13.8 | N13.8 | N13.801 |
| 6. Other related diagnosis |  |  |  |  |
| Hereditary nephropathy, not elsewhere classified |  | N07 | N07.901 | N07 |
| Glomerular disorders in diseases classified elsewhere | N08, exclude N08.5 |  |  |  |
| Renal agenesis and other reduction defects of kidney | Q60 |  |  |  |
| Polycystic kidney, autosomal recessive | Q61.1 |  |  |  |
| Polycystic kidney, autosomal dominant | Q61.2 |  |  |  |
| Polycystic kidney, unspecified | Q61.3 |  |  |  |
| Medullary cystic kidney, sponge kidney NOS | Q61.5 |  |  |  |
| Lobulated, fused and horseshoe kidney | Q63.1 |  |  |  |
| Congenital malformation of kidney, unspecified | Q63.9 |  |  |  |
| Gout due to impairment of renal function |  | M10.300 | M10.393 | M10.300 |
| Unspecified contracted kidney | N26 |  |  |  |
| Ischemia and infarction of kidney | N28.0 |  |  |  |
| Other specified disorders of kidney and ureter | N28.8 |  |  |  |
| Disorders of kidney and ureter, unspecified | N28.9 |  |  |  |
| Congenital renal failure |  | P96.0 | P96.0 | P96.000 |
| Extrarenal uremia | R39.2 |  |  |  |
| Aortic arch syndrome + renovascular hypertension |  | M31.4 + I15.0 | I77.604 + I15.0 | I77.600x004 + I15.0 |
| Goodpasture syndrome | M31.001 |  |  |  |
| Renal osteodystrophy | N25.0 |  |  |  |
| Failure and rejection of renal transplantation | T86.1 |  |  |  |
| Hemolytic uremic syndrome | D59.3 |  |  |  |
| Dialysis | Z49 |  |  |  |
| Renal allergic purpura | D69.005+N08.2 |  |  |  |
| Lupus nephritis |  | M32.101+N08.5 | M32.105+N08.5 | M32.101+N08.5 |
| Goodpasture syndrome-related glomerulonephritis |  | M31.003+N08.5 |  | M31.003+N08.5 |
| Antiglomerular basement membrane antibody-related disease |  | M31.002+N08.5 | M31.005+N08.5 | M31.002+N08.5 |
| Microscopic polyangitis |  | M31.700 | M31.701 | M31.700 |
| ANCA-related nephritis |  | M31.701+N08.5 | M31.802 | M31.701+N08.5 |
| Thrombotic thrombocytopenic purpura-related glomerulonephritis |  | M31.102+N08.5 |  | M31.102+N08.5 |
| Wegener’s granulomatosis-related glomerulonephritis |  | M31.303+N08.5 |  | M31.303+N08.5 |
| Pregnancy with nephrotic syndrome |  | O26.801 | O26.811 | O26.801 |
| Pregnancy with glomerulonephritis |  | O26.804 | O26.812 | O26.804 |
| Pregnancy with renal failure |  | O26.802 | O26.813 | O26.802 |
| HBV-related nephritis |  | B18.103+N08.0 | B18.102 | B18.103+N08.0 |
| HCV-related nephritis |  | B18.205+N08.0 | B18.208 | B18.205+N08.0 |
| Cryoglobulinaemia-related glomerulonephritis |  | D89.101+N08.2 |  | D89.101+N08.2 |
| Hereditary amyloidosis nephropathy |  | E85.002 | E85.003 | E85.002 |
| Amyloidosis-related nephropathy |  | E85.411+N29.8 | E85.410+N08.4 | E85.411+N29.8 |
| Psoriatic nephritis |  | L40.803+ | L40.802+N05.9 | L40.800x002+N05.9 |
| Kidney injury-related gout |  | M10.300 | M10.393 | M10.300 |
| Syphilitic nephritis |  |  | A52.712+N08.0 | A52.700x012+N08.0 |
| Lupus kidney injury |  |  | M32.112+N08.5 |  |
| Lupus nephritis |  | M32.101+N08.5 | M32.105+N08.5 | M32.101+N08.5 |
| Lupus tubulointerstitial kidney |  | M32.102+N16.4 | M32.113+N16.4 | M32.102+N16.4 |
| Gouty nephropathy |  |  | M10.391 | M10.300x091 |
| Gouty nephrolithiasis |  | M10.005+N22.8 | M10.392 | M10.005+N22.8 |

**Table S2. Descriptive statistics of predictor variables for intercity mobility of CKD patients.**

| Feature type | Feature name | Meaning | Unit | Median [IQR] | Data source |
| --- | --- | --- | --- | --- | --- |
| Socio-economic condition | AAP | Average annual population | million | 3.79 [2.39, 5.89] | *Landscan* *2015* |
|  | NGR | Natural population growth rate | ‰ | 3.10 [2.15, 4.56] |  |
|  | PPA | Proportion of the population aged 60 and over | % | 17.3 [17.1, 17.4] | *China Statistical Yearbook* |
|  | GDP | City’s contribution to gross domestic product per capita | ¥1000 | 43.6 [28.1, 60.4] | *China City Statistical Yearbook 2015* |
|  | GDPGR | *GDP* growth rate | % | 8.70 [7.38, 9.85] |  |
|  | UEBPI | Urban Employee Basic Pension Insurance coverage proportion | % | 16.3 [11.0, 27.6] |  |
|  | UBMI | Urban Basic Medical Insurance coverage proportion | % | 17.9 [11.4, 29.4] |  |
| Medical resources | NHH | Number of hospitals | pmp | 42.6 [33.5, 56.7] | *China City Statistical Yearbook 2015* |
|  | NHHB | Number of hospital beds | pmp | 4394 [3680, 5267] |  |
|  | NOD | Number of licensed doctors | pmp | 2046 [1605, 2718] |  |
|  | NNP | Number of nephrologists | pmp | 5.41 [2.56, 10.14] | *Haodaifu* (www.haodf.com)*, WeDoctor* (www.guahao.com) |
|  | PON | Proportion of nephrologists per thousand doctors | ‰ | 2.86 [1.44, 4.52] | - |
| Traffic convenience | TEC | Traffic eigenvector centrality | - | 0.09 [0.00, 2.92] | OpenFlights and OpenStreetMap datasets |

**Table S3. Covariates included in each Geographically Weighted Regression (GWR) model following variance inflation factor (VIF) screening.**

| Dependent variable | Independent variable | Covariates |
| --- | --- | --- |
| CKD-related hospitalization outflows | NHHB | GDP+NHH+GDPGR+PON+AAP+TEC+UEBPI+PPA |
|  | NGR | NHHB+GDP+NHH+GDPGR+PON+TEC+UEBPI+PPA |
|  | NOD | GDP+NHH+GDPGR+PON+AAP+TEC+UEBPI+PPA |
| CKD-related hospitalization inflows | PPA | NHHB + GDP + NOD + NHH + GDPGR + PON + AAP + TEC |
|  | PON | NHHB + GDP + NOD + NHH + GDPGR + AAP + TEC + PPA |
|  | NNP | NHHB + GDP + NHH + GDPGR + AAP + TEC + PPA |
| AAP, Average annual population; NGR, Natural population growth rate; GDP, City’s contribution to gross domestic product per capita; GDPGR, GDP growth rate; UEBPI, Urban Employee Basic Pension Insurance coverage proportion; UBMI, Urban Basic Medical Insurance coverage proportion; NHH, Number of hospitals; NHHB, Number of hospital beds; NOD, Number of licensed (assistant) doctors; NNP, Number of nephrologists; PON, Proportion of nephrologists per thousand doctors; TEC, Traffic eigenvector centrality; PPA, Proportion of the population aged 60 and over. | | |


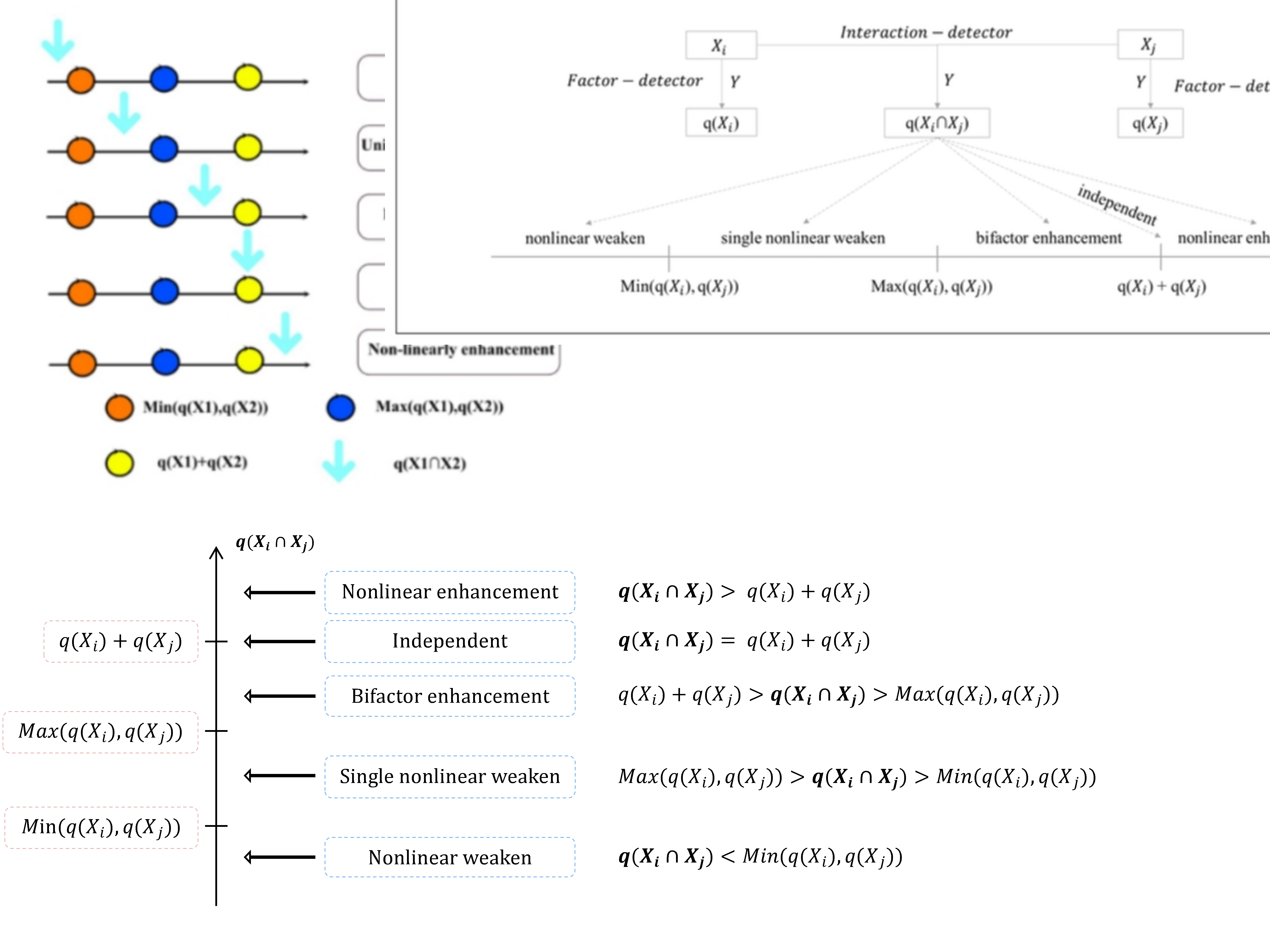


**Figure S1. The interactive patterns of two factors identified by Geodetector model.**


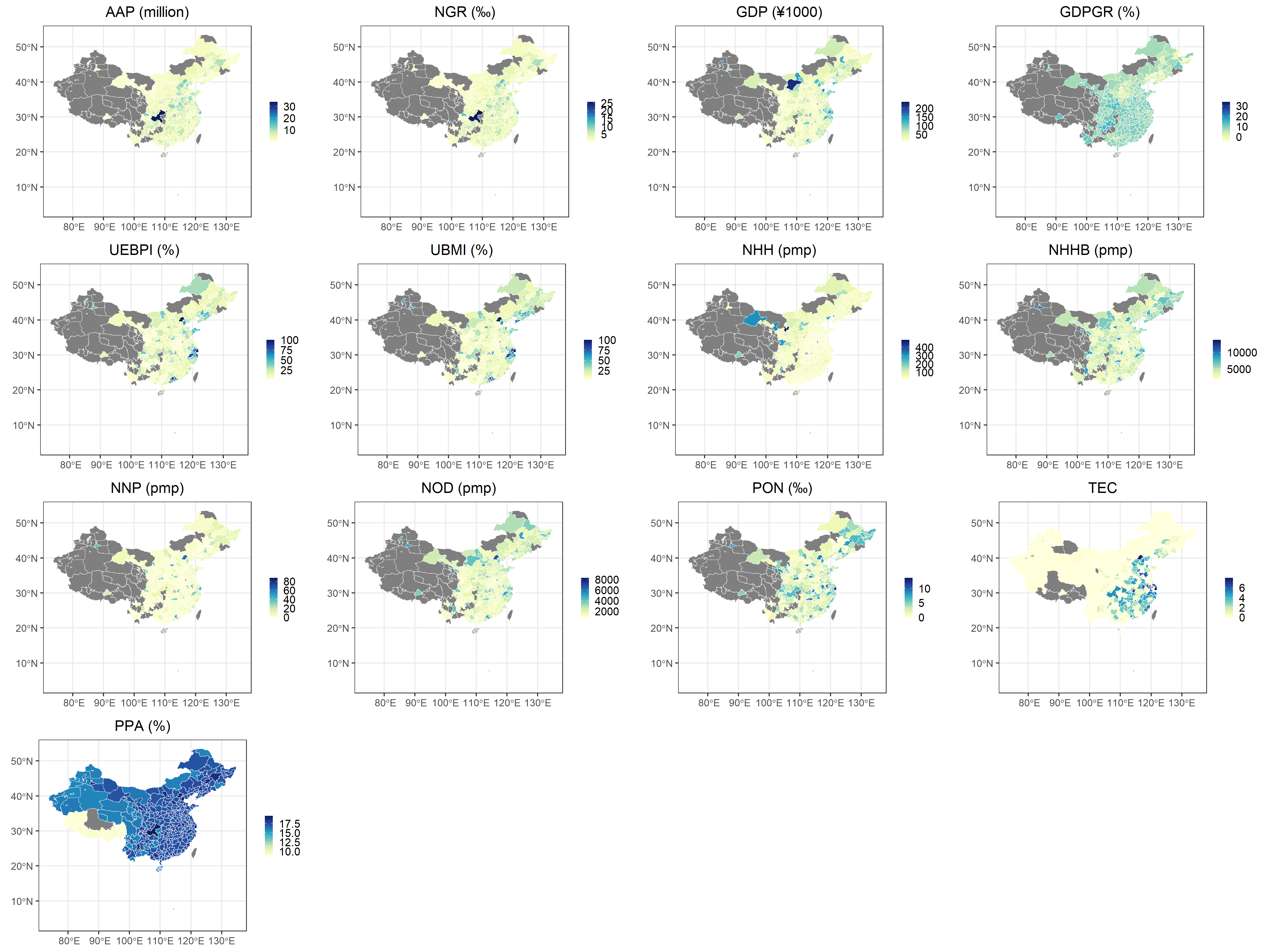


**Figure S2. The spatial distributions of socioeconomic, healthcare resource, and transportation factors across cities in China.** The figure displays the geographical variations of thirteen key independent variables used in the study. Each panel represents a specific variable, with colors indicating the intensity or value of the indicator; darker blue shades correspond to higher values, while lighter yellow shades indicate lower values. Gray areas represent regions with missing data. The map data were obtained from the National Geomatics Center of China. AAP, Average annual population; NGR, Natural population growth rate; GDP, City’s contribution to gross domestic product per capita; GDPGR, GDP growth rate; UEBPI, Urban Employee Basic Pension Insurance coverage proportion; UBMI, Urban Basic Medical Insurance coverage proportion; NHH, Number of hospitals; NHHB, Number of hospital beds; NOD, Number of licensed (assistant) doctors; NNP, Number of nephrologists; PON, Proportion of nephrologists per thousand doctors; TEC, Traffic eigenvector centrality; PPA, Proportion of the population aged 60 and over.

a)


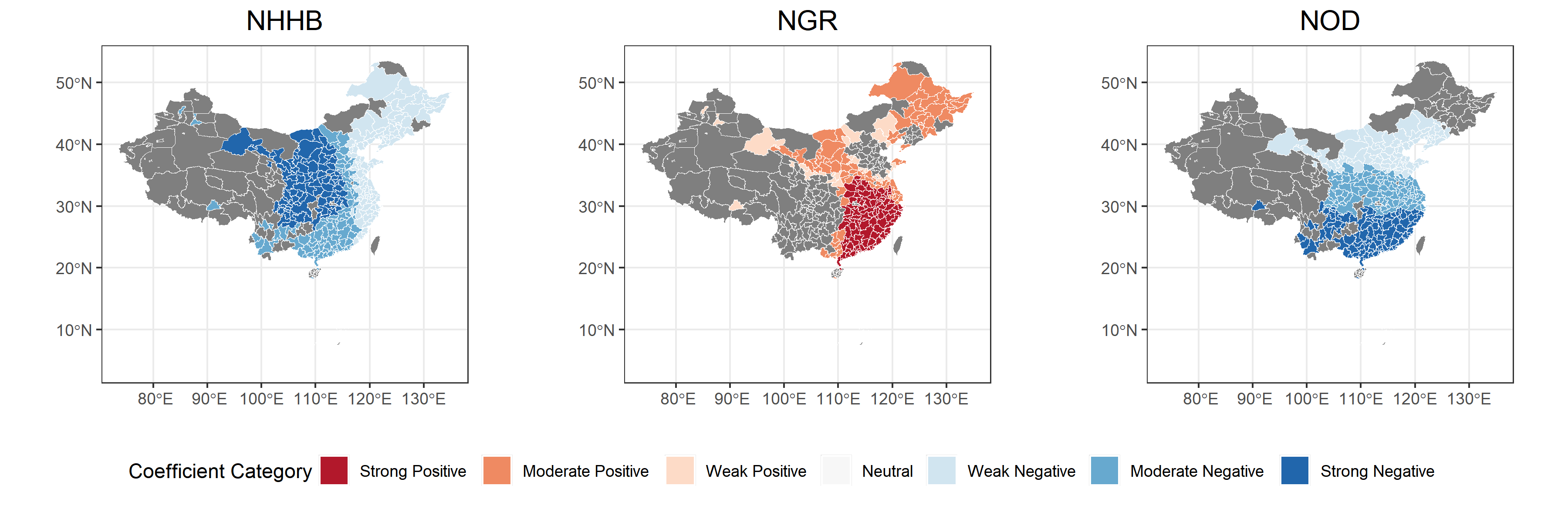


b)


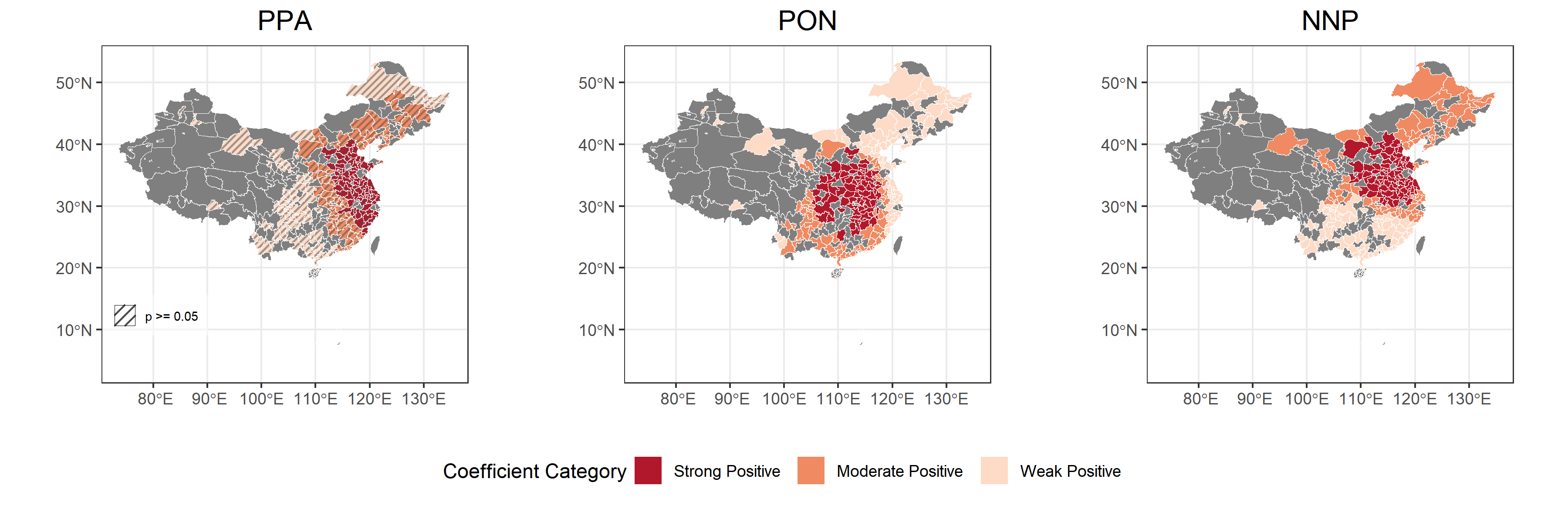


**Figure S3.** **Sensitivity analysis of spatial distribution of the local parameter estimates for the key influencing factors estimated using the MGWR model, with top interaction terms identified by Geodetector.** a) Outflow hospitalizations models showing spatial variation in the influence of number of hospital beds (NHHB), natural population growth rate (NGR), and number of licensed doctors (NOD). Only statistically significant local parameter estimates from the MGWR models are presented. b) Inflow hospitalizations models showing spatial variation in the influence of proportion of the population aged 60 and over (PPA), proportion of nephrologists per thousand doctors (PON), and number of nephrologists (NNP), and number of hospital beds (NHHB). Hatched areas indicate local estimates that are not statistically significant (*P* > 0.05). Colors represent the direction and strength of the coefficients: Red shades indicate positive associations, categorized as dark red (Strong Positive), orange (Moderate Positive), and light peach (Weak Positive). Blue shades indicate negative associations, categorized as dark blue (Strong Negative), medium blue (Moderate Negative), and light blue (Weak Negative). Coefficients were classified into these six categories using tertile thresholds: Strong, Moderate, and Weak Negative for values below 0; and Weak, Moderate, and Strong Positive for values above 0. Gray areas represent regions with missing data. Map data source: National Geomatics Center of China.


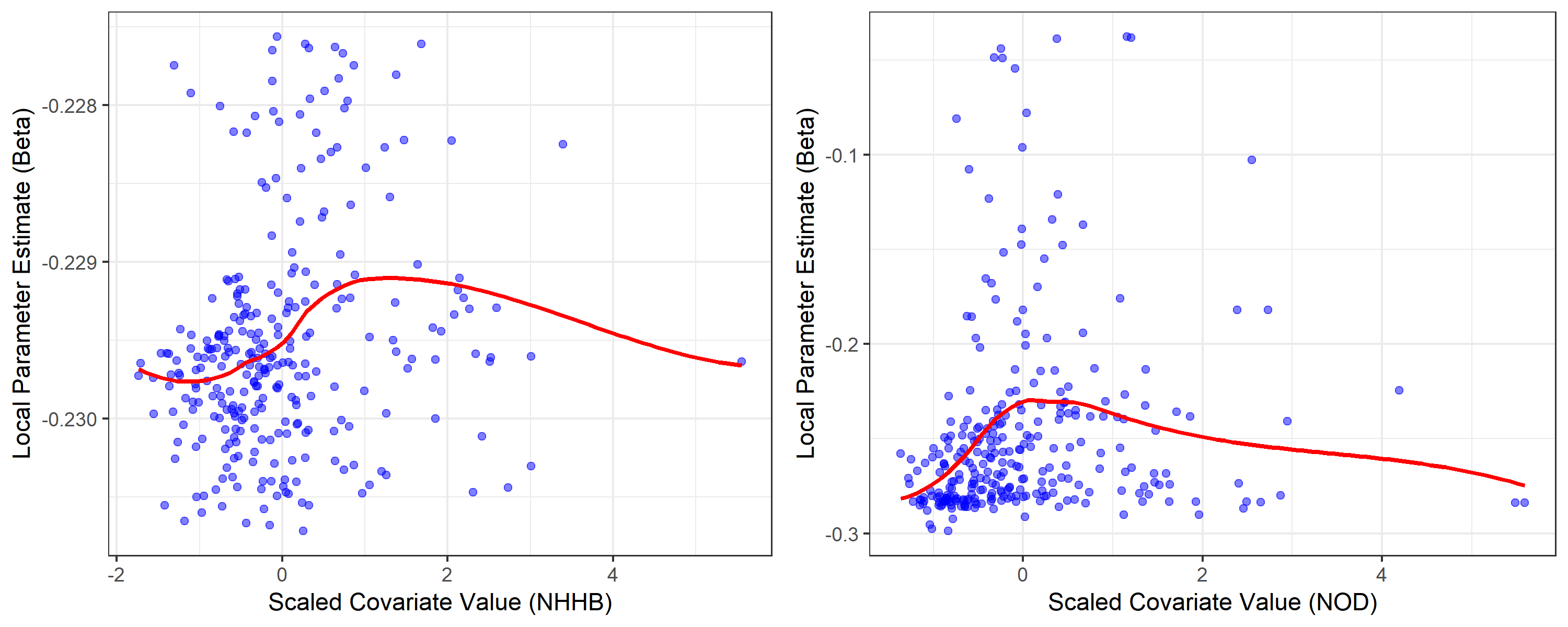


## Figure S4. Diagnostic screening for global non-linearity and spatial heterogeneity of NHHB and NOD in the patient mobility outflow model. The scatter plots illustrate the relationship between the standardized covariate values (x-axis) and the local parameter estimates (y-axis) to identify potential non-linear associations. Blue circles represent the parameter estimates for individual cities, while the solid red line indicates the fitted smoothing curve. Covariate values are standardized to z-scores (mean = 0, standard deviation = 1). NHHB, Number of hospital beds; NOD, Number of licensed (assistant) doctors

a)


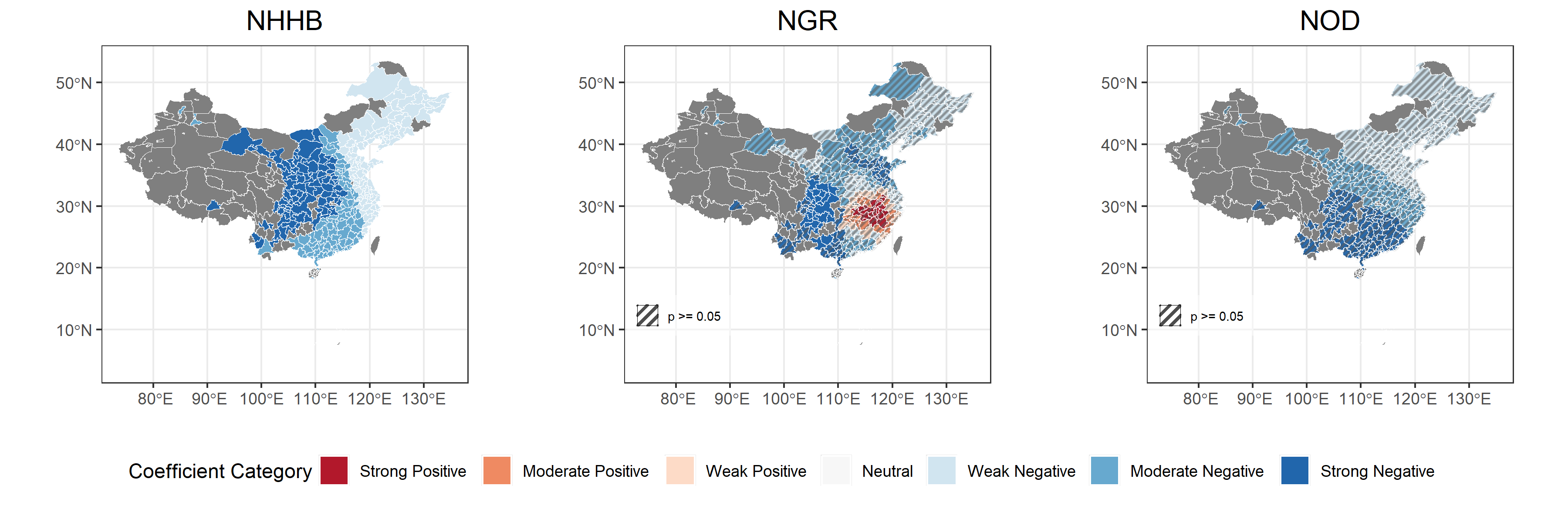


b)


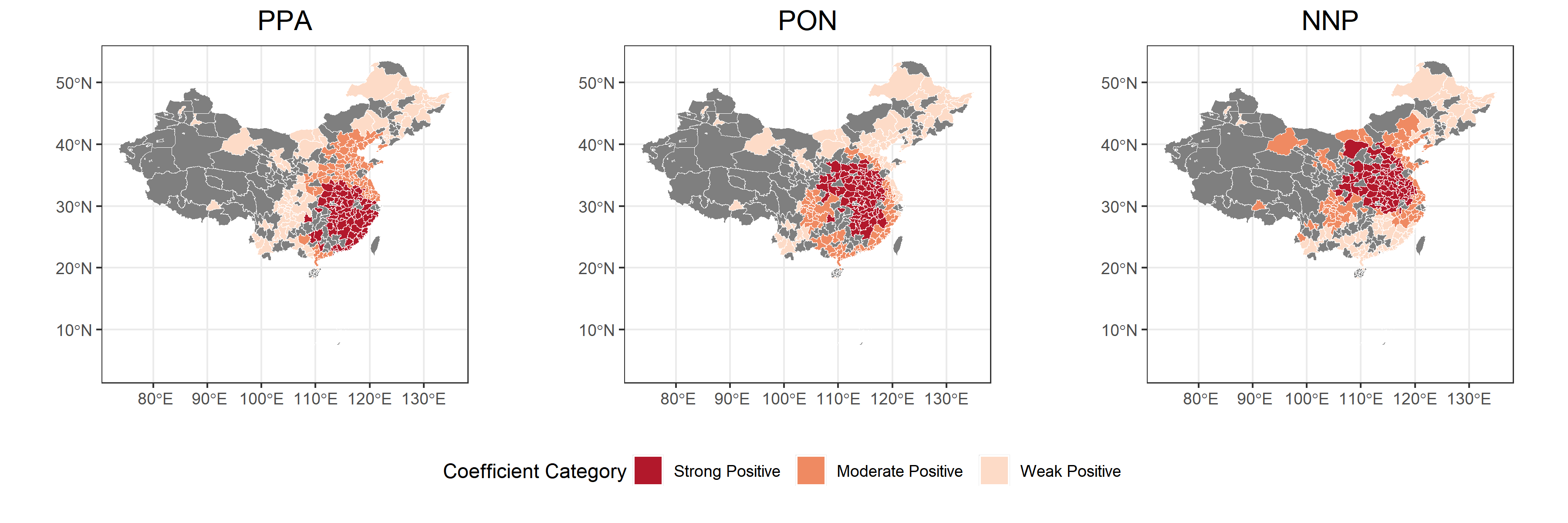


**Figure S5. Sensitivity analysis of spatial distribution of local parameter estimates for key influencing factors in the MGWR model including all covariates.** a) Outflow hospitalizations models showing spatial variation in the influence of number of hospital beds (NHHB), natural population growth rate (NGR), and number of licensed doctors (NOD). b) Inflow hospitalizations models showing spatial variation in the influence of proportion of the population aged 60 years and over (PPA), proportion of nephrologists per thousand doctors (PON), and number of nephrologists (NNP). For inflow models, PON and NNP were alternately excluded to avoid multicollinearity. Hatched areas indicate local estimates that are not statistically significant (*P* > 0.05). Colors represent the direction and strength of the coefficients: Red shades indicate positive associations, categorized as dark red (Strong Positive), orange (Moderate Positive), and light peach (Weak Positive). Blue shades indicate negative associations, categorized as dark blue (Strong Negative), medium blue (Moderate Negative), and light blue (Weak Negative). Coefficients were classified into these six categories using tertile thresholds: Strong, Moderate, and Weak Negative for values below 0; and Weak, Moderate, and Strong Positive for values above 0. Gray areas represent regions with missing data. Map data source: National Geomatics Center of China.
